# Additive Biomanufacturing: An Advanced Approach for Periodontal Tissue Regeneration

**DOI:** 10.1007/s10439-016-1687-2

**Published:** 2016-07-29

**Authors:** Sarah-Sophia D. Carter, Pedro F. Costa, Cedryck Vaquette, Saso Ivanovski, Dietmar W. Hutmacher, Jos Malda

**Affiliations:** 1 Department of Orthopedics, University Medical Center Utrecht, Utrecht, The Netherlands; 2 Biofabrication Facility, Regenerative Medicine Center Utrecht, University Medical Center Utrecht, Utrecht, The Netherlands; 3 Institute of Health and Biomedical Innovation, Queensland University of Technology, Brisbane, Australia; 4 School of Dentistry and Oral Health, Griffith University, Gold Coast, Australia; 5 Menzies Health Institute Queensland, Griffith University, Gold Coast, Australia; 6 Institute for Advanced Study, Technical University Munich, Munich, Germany; 7 Department of Equine Sciences, Faculty of Veterinary Medicine, Utrecht University, Utrecht, The Netherlands

**Keywords:** Periodontology, Guided tissue regeneration, Cell sheet engineering, Tissue engineering & regenerative medicine (TE&RM), Nanotechnology, Electrospinning, Scaffolds, Bioprinting

## Abstract

Periodontitis is defined as a chronic inflammatory condition, characterized by destruction of the periodontium, composed of hard (i.e. alveolar bone and cementum) and soft tissues (i.e. gingiva and periodontal ligament) surrounding and supporting the teeth. In severe cases, reduced periodontal support can lead to tooth loss, which requires tissue augmentation or procedures that initiate a repair, yet ideally a regenerative response. However, mimicking the three-dimensional complexity and functional integration of the different tissue components *via* scaffold- and/or matrix-based guided tissue engineering represents a great challenge. Additive biomanufacturing, a manufacturing method in which objects are designed and fabricated in a layer-by-layer manner, has allowed a paradigm shift in the current manufacturing of medical devices and implants. This shift from design-to-manufacture to manufacture-to-design, seen from a translational research point of view, provides the biomedical engineering and periodontology communities a technology with the potential to achieve tissue regeneration instead of repair. In this review, the focus is put on additively biomanufactured scaffolds for periodontal applications. Besides a general overview of the concept of additive biomanufacturing within this field, different developed scaffold designs are described. To conclude, future directions regarding advanced biomaterials and additive biomanufacturing technologies for applications in regenerative periodontology are highlighted.

## Introduction

Periodontitis is a highly prevalent disease caused by a bacterial biofilm.^40^ It is defined as a chronic inflammation resulting in irreversible destruction of the periodontium, which consists of the hard (i.e. alveolar bone and cementum) and soft (i.e. gingiva and periodontal ligament) tissues surrounding and supporting the teeth. In severe cases, reduced periodontal support can lead to tooth loss, requiring tissue augmentation or regenerative procedures to restore the lost function and appearance.

Over the last four decades, various attempts have been made to achieve regeneration instead of repair of the periodontal apparatus. These include root surface conditioning, graft materials, barrier membranes, gene therapy, and growth factors.^17^ However, all of the above-mentioned approaches are still associated with significant clinical drawbacks; the availability of autologous grafts is limited, gene therapy may trigger host immune reactions or tumorigenesis, growth factors are often unstable, and biomaterials are linked with a high failure rate. Hence, there is a significant need for treatments with high efficacy and efficiency, paving the way for periodontal tissue restoration.

Recent developments in the science, technology, engineering, and mathematics (STEM) arena allow for alternatives to address several challenges in the treatment of periodontal disease. An example of such an advancement is tissue engineering, which aims to regenerate tissues by combining cells with scaffolds and bioactive factors.^36^ It is noteworthy that in contrast to periodontal repair, which refers to healing without full reconstitution of the lost tissues, periodontal regeneration aims at restoring the structure and function of the periodontal complex in its entirety.^5^ Nevertheless, guiding the regeneration of the complex architecture of the periodontium still represents one of the greatest challenges in modern dentistry. The functional integration of scaffolds and/or matrices, which synchronously guide the regeneration of the soft and hard tissues, is demanding from both an anatomical and a physiological perspective.

Over the years, the field of tissue engineering has made tremendous progress. Third generation biomaterials and advanced processing technologies have enabled a shift in the current manufacturing concept, resulting in scaffolds with highly tailored properties for challenging applications, including functional and load-bearing ones. This paradigm shift from design-to-manufacture to manufacture-to-design includes the integration of medical imaging with additive biomanufacturing techniques; enabling manufacturing of highly personalized medical devices and implants. However, there is a strong, unmet demand for hybrid and multiphasic materials that functionally combine spatial, mechanical, and biological benefits. In the context of periodontal regeneration, additive biomanufacturing, which referrers to the translation of additive manufacturing technologies into the field of tissue engineering and regenerative medicine (TE&RM), has significant advantages.^8^ This approach, in which three-dimensional (3D) structures are fabricated in a layer-by-layer manner based on a computer-aided design (CAD), facilitates the development of multiphasic scaffolds. These multiphasic scaffolds consist of a hierarchical architecture to guide simultaneous tissue formation, which could further mimic the properties and architectural configuration of periodontal tissues.^24^^,^ 35

In this review, the role of additive biomanufacturing within the field of periodontal regeneration is analyzed and discussed. After a brief description of the concept of periodontal therapy, relevant additive biomanufacturing technologies for applications within the field of periodontal regeneration are highlighted. Special emphasis is put on the design of currently available scaffolds. To bring the review to an end, future directions in the context of advanced biomaterials and additive biomanufacturing technologies for regenerative periodontology are described.

## Historical Perspective of Periodontal Therapy

**Figure 1:**
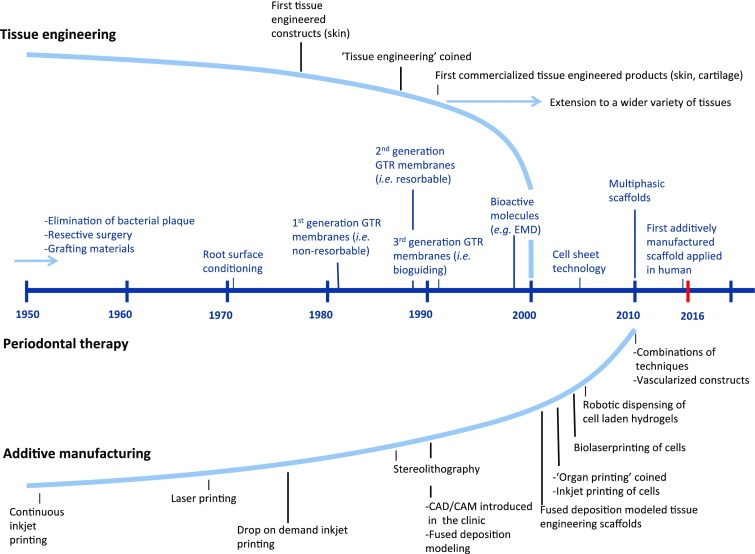
Key developments within the fields of tissue engineering and additive (bio)manufacturing and their applications in periodontal therapy.^2^^,^ 4 ^,^ 31 Early approaches to periodontal therapy were mainly focused on mechanical control of the biofilm and resective surgeries. With the realization that ingrowth of gingival epithelium cells during periodontal wound healing limits new periodontal attachment formation, the surgical technique GTR was developed.^2^ The first generation of GTR membranes involved the use of non-resorbable materials (e.g. expanded polytetrafluoroethylene). To circumvent the need for additional surgery to remove these membranes, resorbable membranes (e.g. collagen) were developed. A more recent approach is based on GTR membranes in combination with bioactive molecules. With the introduction of tissue engineering and additive (bio)manufacturing within the field of periodontology around 2000 and 2010 respectively, novel approaches, such as cell sheet engineering and (multiphasic) scaffolds, were developed.^24^^,^ 42 ^,^ 43 ^,^ 46

As described by Bartold *et al*., over the past 30 years major advances have been made within the field of periodontal therapy.^2^^,^ 23 Yet, a main drawback of early attempts to achieve periodontal tissue restoration, such as grafting materials and guided tissue regeneration (GTR), was the unpredictable clinical outcome. As for any field to advance, progressions within converging disciplines play an important role. Pillars for the development of currently available scaffolds include tissue engineering and additive (bio)manufacturing (Fig. 1). In the early 2000s, the concept of tissue engineering was introduced within the field of periodontology.^3^ By combining cells with scaffolds and bioactive factors, more controlled repair and/or regeneration of tissues was envisioned.^35^ Along with the increasing knowledge of the biological principles underlying periodontitis and the development of novel regenerative approaches, different manufacturing methods were considered. While additive (bio)manufacturing in the clinic was already introduced in the 1990s, this technology was initially mainly centered on the development of surgical tools and customized models, useful in the pre-operative planning of surgeries.^2^^,^ 23 However, the ability to create constructs with precisely defined properties and geometries in an automated and reproducible manner make this approach interesting for production of tissue engineering scaffolds. As shown below, in currently available scaffolds both the concepts of tissue engineering and additive (bio)manufacturing converge.

### Cell Sheet Engineering

**Figure 2:**
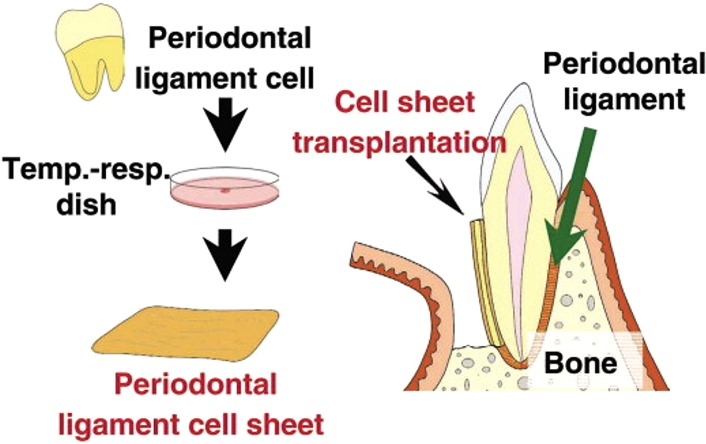
Cell sheet technology for periodontal tissue regeneration. After isolation of the desired cells and cell sheet formation, the cell sheets are physically harvested and transplanted into the side of the defect. Figure obtained from Yamato and Okano.^46^ Reproduced with permission.

An example that illustrates the emergence of tissue engineering within the field of periodontology is cell sheet engineering. This concept, which has been playing an increasingly significant role in currently available constructs aiming at periodontal regeneration, involves thermo-responsive surfaces [e.g. poly(*N*-isoproplyacrylamide) (PIPAAm)] enabling non-enzymatic harvesting of cells and deposited extracellular matrix (ECM).^45^ At cell culture conditions of 37°C, PIPAAm is relatively hydrophopic, facilitating attachment of cells to the surface. Below the lower critical solution temperature of 32°C, PIPAAm becomes relatively hydrophilic, enabling detachment of cells without the need for proteolytic treatments. Regarding restoration of the periodontal apparatus, after tissue extraction and isolation of the cells facilitating periodontal regeneration, a cell sheet is formed *in vitro* by stimulating the deposition of ECM. Subsequently, the cell sheet is transplanted into the periodontal defect, enabling enhanced control over cell delivery (Fig. 2).^22^

Cell sheet technology has already shown promising results in terms of periodontal regeneration potential in (pre)clinical trials.^1^^,^ 15 ^,^ 19 ^,^ 25 ^,^ 26 However, a drawback associated with this approach is the lack of biomechanical stability. Furthermore, stabilizing and securing the cell sheet within the complex-shaped periodontal defect is challenging. Attempts to improve the biomechanical properties of cell sheets include layering of several sheets, supporting the sheets with hydrogels, and addition of ECM components to the thermo-responsive polymer.^16^^,^ 26 In addition, several groups explored the combination of cell sheets with additively biomanufactured carrier constructs. Dan *et al*. showed the potential of porous polycaprolactone (PCL) scaffolds to promote periodontal regeneration.^11^ In this study, a calcium phosphate (CaP)-coated PCL monophasic scaffold was manufactured as a carrier for different periodontal cell sheets (i.e. human periodontal ligament cells, gingival margin-derived cells, and alveolar bone-derived cells). *In vivo* evaluations revealed a significant promotion of periodontal attachment formation when using the periodontal ligament cell sheets in combination with the carrier membrane. In addition, although to a limited extent, cell sheets prepared from alveolar bone-derived cells showed to promote this attachment. Another approach involving a PCL membrane to support the cell sheet was presented by Farag *et al*.^13^ In this complementary approach, decellularized human periodontal ligament cell sheets were examined for periodontal regeneration. The rationale behind this approach was the capability of the cell sheet to maintain ECM integrity, allowing recellularization with allogenic cells.

## Additive Biomanufacturing Applied to Periodontal Scaffold Design and Fabrication

The design of scaffolds which mimic the complex periodontal shape and organization represents a significant challenge in regenerative periodontology. Although additive biomanufacturing may help to surmount this hurdle, adoption and long-term success of these strategies greatly rely on the biomaterials being used. Regarding periodontal regeneration, the most commonly used materials for restoring and/or replacing lost oral tissues are ceramics and polymers (Table 1).^48^ Ceramic biomaterials such as CaP, calcium sulfate (CS), and bioactive glass (BG) are ideal candidates for hard-tissue engineering and restoring the lost function due to their similar composition to bone mineral, the stimulating effects on cell proliferation and differentiation, and their relatively low degradation rate, the latter specifically facilitating prolonged guided tissue remodeling and structural support. Despite these advantages, the brittleness and low ductility need to be considered when using these materials. Synthetic polymers on the other hand, such as polylactic acid (PLA), polyglycolic acid (PGA), the copolymer poly(lactic-co-glycolic acid) (PLGA), and PCL have highly adjustable characteristics, excellent production repeatability, and can potentially be mass produced. However, the process of printing synthetic polymers involves using parameters detrimental to cell viability (e.g. high temperature), making the incorporation of cells and growth factors into the polymer mixture complicated if not impossible.^21^

**Table 1:** Commonly utilized biomaterials for periodontal tissue regeneration and their main advantages and disadvantages.

Material		Advantages	Disadvantages
Ceramics	Calcium phosphate (hydroxyapatite and tricalcium phosphate)	• Similar composition to bone mineral • Osteoconductive • Approval for clinical application • Able to stimulate bone healing	• Not compatible with cell encapsulation • Brittleness • Low ductility • Inconsistent cell reactions resulting from variations in surface properties
Synthetic polymers	Polycaprolactone (PCL)	• Highly adjustable material properties (e.g. physiochemical and mechanical) • Wide range of degradation and resorption kinetics • Good production repeatability • Approval for clinical application	• Low bioactivity
	Polyglycolic acid (PGA)		
Natural polymers	Collagen	• Good biocompatibility and cell affinity • Approval for clinical application	• Fast degradation rate • Low mechanical properties

**Figure 3:**
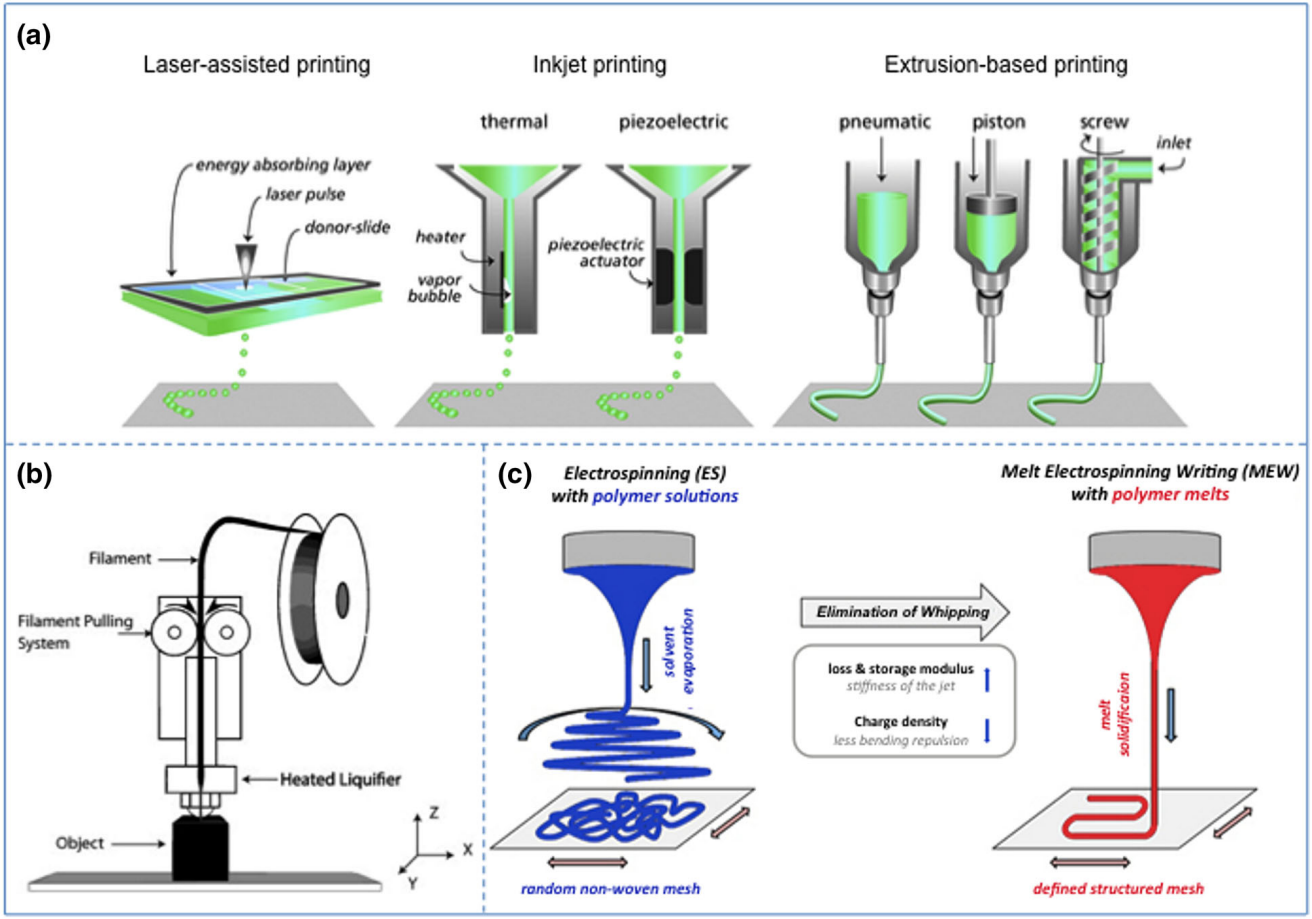
Additive (bio)manufacturing. (a) Main additive (bio)manufacturing techniques; Laser-assisted printing (e.g. laser-induced forward transfer), inkjet printing, and extrusion-based printing (adapted from Malda *et al.* 30) (b) Principle of fused deposition modeling (adapted from Carneiro *et al.* 7) (c) Solution electrospinning and melt electrospinning (adapted from Hochleitner *et al*.^20^). Reproduced with permission.

A wide range of different additive (bio)manufacturing technologies is described in the literature. These can be categorized into laser-assisted printing, inkjet printing, and extrusion-based printing (Fig. 3a). Common to all these technologies is the use of CAD software or digital images for the design.^12^ Extrusion-based printing is the most widely applied technique for potential application in periodontal regeneration. While a great variety of extrusion-based printers are described in the literature, several general characteristics can be identified. These include the temperature-controlled material handling, dispensing system and stage, and an optional light source and piezoelectric humidifier.^32^ As the name indicates, extrusion printing involves the controlled extrusion of a material through a printer head onto a collector. In most of these systems mechanical movement (piston or screw) or a pneumatic system enables the extrusion of the polymer melt or ink leading to the deposition of a filament or strut, which dimensions can be adjusted by modifying the printing conditions (e.g. temperature, feed-rate, velocity of the collector). An example of an extrusion-based printing technique evaluated for periodontal applications is fused deposition modeling (FDM) (Fig. 3b; Table 2). In FDM systems, a thermoplastic material is fed from a filament coil and inserted into a heated nozzle head that enables the deposition of semi-molten state polymer struts onto a substrate.^47^

**Table 2:** Currently applied scaffold fabrication techniques for periodontal regeneration and their main advantages and disadvantages.

Fabrication technique	Working principle	Advantages	Disadvantages
Fused deposition modeling (FDM)	Thermoplastic material is fed from a filament coil, inserted into a heated FDM extrusion head, and deposited on the collector platform	• Layer resolution up to ± 20 micron • No solvents or chemical post-processing required • Low-cost equipment	• Low *Z*-axis resolution • Extruding conditions limit biomedical applications (e.g. high temperature)
Solution electrospinning (SE)	Polymer solution is drawn towards a collector plate with opposite polarity by means of an electrical field	• Production of nanofibers • Small pore diameter^a^	• Need for (toxic) solvents limits biomedical applications • Chaotic fiber deposition • Lack of reproducibility • Costly approach compared to ME
Melt electrospinning (ME)	Polymer melt is drawn towards a collector plate with opposite polarity by means of an electrical field	• In general production of microfibers • No solvents required • Controlled fiber placement and accurate stacking • Reproducible fabrication technique • Lower manufacturing costs when compared to SE	• High processing temperature limits biomedical applications

^a^ Advantageous for specific applications (e.g. filter purposes)

Another scaffold fabrication method explored for periodontal applications is electrospinning, a broadly used micro- to nanofiber fabrication technique.^10^ A typical electrospinning setup consists of a syringe that contains the desired polymer, a syringe pump, a high voltage supply, and a collector plate. Electrospinning can be performed with both polymer solutions and polymer melts, also referred to as solution electrospinning and melt electrospinning respectively (Fig. 3c; Table 2). In solution electrospinning, a polymer solution is extruded through a spinneret and electrified leading to the formation of a jet. Under the influence of the electrical field applied between the spinneret and the collector, the jet undergoes several physical instabilities inducing a whipping and oscillating motion as it travels towards the collector. The high frequency of the oscillation results in a drastic reduction of the jet diameter, which enables the deposition of micro- to nanofibers. On the other hand, the higher viscosity of polymer melts diminishes the electrical instabilities and allows for controlled fiber placement. Since melt electrospinning allows direct writing of the polymer melt, this method can be considered as an additive (bio)manufacturing technique.^6^

## Multiphasic Scaffolds for Periodontal Regeneration

An important aspect of appropriate periodontal regeneration is the establishment of new fiber attachment to the tooth root surface. This is mediated by the formation of neo-cementum onto the tooth surface, allowing for the insertion of the fibers. Several studies have shown that periodontal ligament tissue, in contrast to gingival connective tissue and alveolar bone, is capable of forming this type of attachment interphase.^27^^,^ 34 The current concept of periodontal regeneration is based on the premise that healthy locally available periodontal cells and/or progenitor cells attracted to the periodontal defect have the potential to facilitate regeneration. However, as mentioned previously, achieving 3D complexity and functional integration of the different soft and hard periodontal tissue components is extremely challenging. As described by Ivanovski *et al*., multiphasic scaffolds for periodontal regeneration incorporate several beneficial features, including occlusive membrane properties, the need for appropriate fiber guiding, and optimization of cell sheet technology.^24^ Essential general considerations for multiphasic scaffolds for periodontal tissue engineering include appropriate spatio-temporal tissue formation and strong cohesion between the different compartments.

### Biphasic Scaffolds

For predictable periodontal regeneration to occur, hierarchical tissue formation with the appropriate interfacial connection is required.^37^ Equally important is establishing sufficient strength and mechanical integrity, which is mainly determined by adequate periodontal ligament fiber orientation and their incorporation into the newly formed tissue.

**Figure 4:**
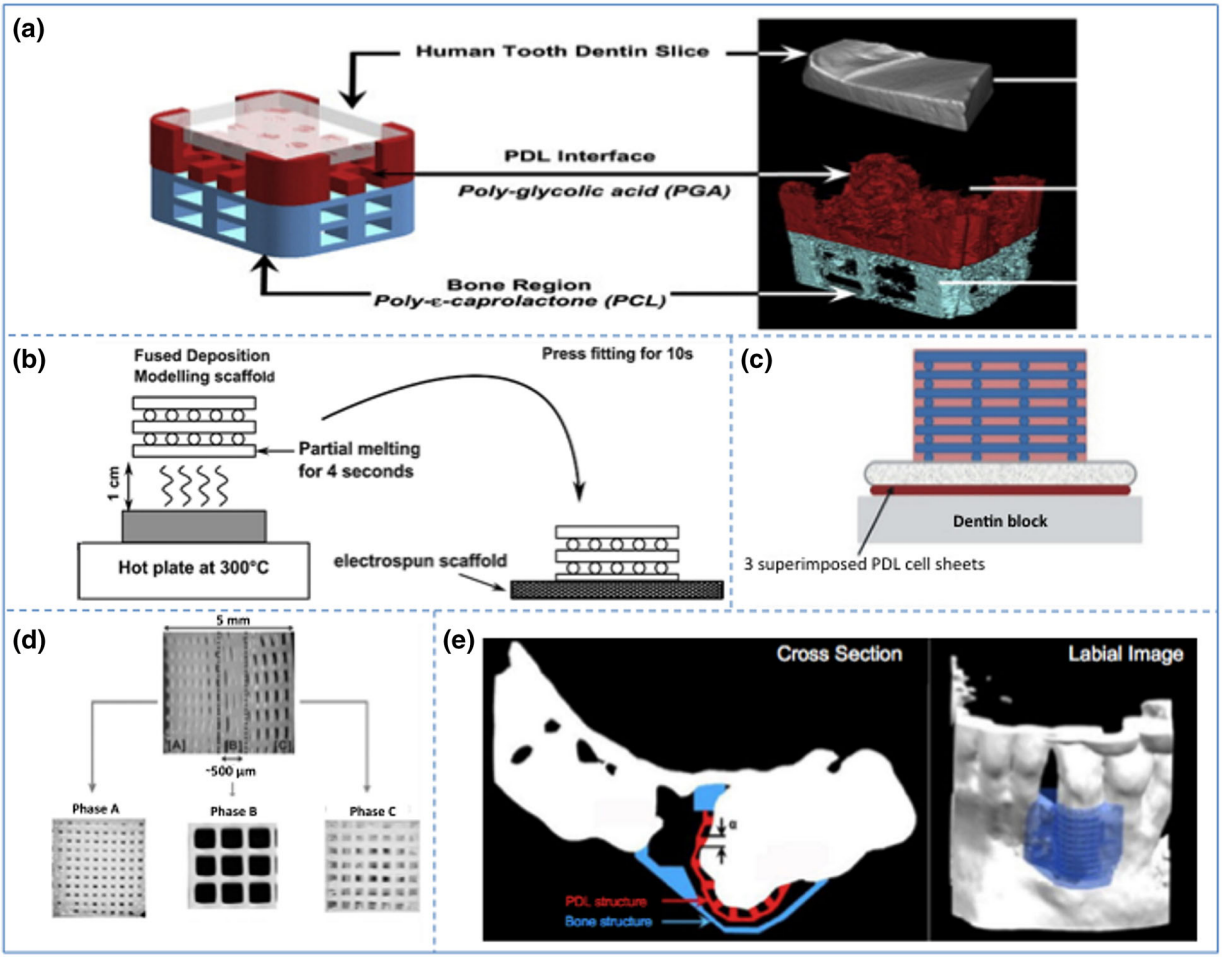
Additively manufactured scaffolds for periodontal regeneration. (a) Biphasic scaffold facilitating fiber orientation (adapted from Park *et al*.^37^) (b) Biphasic scaffold in combination with cell sheet technology (adapted from Vaquette *et al*.^44^) (c) Enhanced biphasic scaffold (adapted from Costa *et al*.^9^) (d) Triphasic scaffold (adapted from Lee *et al.* 29) (e) First additively biomanufactured scaffold for periodontal regeneration applied in human (adapted from Rasperini *et al*.^41^). Reproduced with permission.

To address these challenges, Park *et al*. developed a biphasic PCL–PGA scaffold fabricated *via* computer-aided manufacturing (Fig. 4a).^37^ The scaffold consisted of both periodontal ligament-specific and bone-specific compartments facilitating formation of human tooth dentin-ligament-bone complexes. In this approach, 3D wax-printing systems facilitated the manufacturing of molds, which were used in the fabrication of the hybrid scaffold. Mold characteristics were judiciously chosen in terms of pore size, channel orientation, and tissue specific compartments. After fabrication, the molds were casted with a PCL or PGA polymer solution. To form a single scaffold structure, both compartments were fused with a thin PCL layer. *In vivo* evaluations of the scaffold design in subcutaneous pockets in mice have shown bone and periodontal ligament regeneration capacity and generation of parallel and obliquely oriented fibers. To further resemble the periodontium, adjustments in the design were made.^37^ This approach has demonstrated control over fiber orientation and facilitates morphogenesis of periodontal tissue. Following on from this strategy, Park *et al*. examined the effect of controlled channel architecture in the scaffold design on the tissue interface.^38^ *In vivo* evaluations of this scaffold in periodontal fenestration defects in athymic rats have shown controlled and predictable periodontal fiber orientation, controlled tissue infiltration, and a better organization of the ligament interface compared with random scaffold architectures. With this image-based, fiber-guiding scaffolding system, the authors intend to predictably facilitate regeneration and integration of dental supporting tissues.^39^ It is noteworthy that although the main focus was put on periodontal applications, the general design with the controlled pore architecture is expected to suit diverse scenarios involving the regeneration of tissue interfaces.

### Additively Manufactured Scaffolds in Combination with Cell Sheet Technology

As described above, another approach aiming at periodontal tissue regeneration involves cell sheet engineering. In addition to utilization of the cell sheets unaided, several groups have investigated the combination of additively manufactured scaffolds with cell sheet technology.

For example, Vaquette *et al*. described a biphasic scaffold in combination with cell sheet technology to regenerate alveolar bone and periodontal ligament simultaneously (Fig. 4b).^44^ The scaffold design was based on a fused deposition modeled component for the bone compartment and a more flexible solution electrospun component for the periodontal ligament compartment. To fabricate the bone compartment, PCL containing β-TCP was utilized. The micro-fibrous membrane facilitating the delivery of periodontal ligament cell sheets consisted of PCL. In order to fuse the different scaffold components, heat treatment was used. Despite promising results in terms of ECM mineralization and enhanced mechanical stability of the cell sheets, the bone compartment did not enable sufficient ectopic bone ingrowth.

Further advancements of this strategy involved an osteoconductive biphasic scaffold devised by Costa *et al.* (Fig. 4c).^9^ In accordance with Vaquette *et al*., a fused deposition modeled bone component and a flexible electrospun periodontal component were taken into account.^44^ To further enhance the osteoconductivity, a CaP coating was deposited on the fused deposition modeled bone compartment. To improve cross-communication between the periodontal ligament and the newly formed alveolar bone, a melt electrospun membrane with a larger pore size was designed. In line with Vaquette *et al*., the scaffold was assembled by using heat treatment.^44^ The adjustments made in the scaffold design significantly increased bone formation and permitted the attachment of functionally oriented periodontal ligament-like tissue and blood vessel ingrowth.

### Triphasic Scaffolds

As an extension of the biphasic scaffolds, Lee *et al*. developed a triphasic scaffold (Fig. 4d).^29^ This approach was based on previous work by Lee *et al*. and involved printing of a seamless scaffold with region-specific pore sizes for the purpose of facilitating integrated regeneration of various tissues.^28^ The scaffold was fabricated by using FDM and consisted of a compartment for the cementum/dentin interface, a compartment for the periodontal ligament, and a compartment for alveolar bone. By combining biophysical properties with spatially released bioactive cues, regeneration of periodontal tissue was envisioned. Although promising, a possible limitation of this approach might be the stiffness of the PCL scaffold, which impedes adaptability to the complex 3D anatomy of different periodontal defects and, hence, limits from a translational research point of view.

Recently, Rasperini *et al*. fabricated the first reported personalized additively biomanufactured scaffold for periodontal osseous defect regeneration in humans (Fig. 4e).^41^ In this study, a customized scaffold was printed by using a computed tomography scan of the patient’s defect. The scaffold, made from PCL powder containing HA, was manufactured by using selective laser sintering technology, which allowed for precise scaffold features, supporting tissue regeneration. Despite the fact that Rasperini *et al*. have demonstrated the potential of this additively manufactured scaffold for the treatment of a large periodontal osseous defects, the scaffold became exposed after 12 months, which ultimately led to failure from a clinical point of view. A more rapidly resorbing biomaterial with a less bulky design would probably have better suited this application. Additionally, the observed limited bone regeneration indicated the need for incorporating scaffold design imperatives, such as larger pores and pore interconnections.

As demonstrated, multiphasic scaffolds facilitate compartmentalized tissue healing, which is essential for periodontal tissue regeneration. In general, multiphasic scaffolds for periodontal regeneration are characterized by the presence of bone and periodontal attachment compartments.^24^ Important characteristics to consider when designing these scaffold are the crucial time-caused steps in the regeneration process, which involve cementum formation onto the root surface, appropriate periodontal fiber formation and insertion into the hard tissues, and sufficient stiffness in general. While the above-described studies seem promising, there is a need for optimization and evaluation in large animal studies and human clinical studies.

## Future Directions

Additive biomanufacturing offers favorable strategies for periodontal tissue engineering. Although encouraging improvements have been made thus far, novel strategies continue to develop at a rapid pace. Besides optimization of the biomaterials, scaffold design, and fabrication techniques, achievement of standardization is an important step towards successful implementation in the future.

### Nanotechnology

Appropriate selection of biomaterials that can guide tissue regeneration plays a vital role in establishing predictable long-term results. For many years, research was focused on the development of biomaterials that simply fill the spaces of the absent tissue.^14^^,^ 33 However, the biomaterial discipline has evolved tremendously and the current trend is to develop materials that significantly direct and guide the biology of the oral environment. Central to recent developments in polymer hybrid materials is the concept of ‘structure-related properties’, which could be considered different from the more established concept of ‘polymer nanocomposites’, the latter focusing on engineering of the functional properties of materials by mixing constituents. Recent advancements in the field of polymer chemistry and plasma physics now facilitate the precise coupling of synthetic polymer and biological- or inorganic-derived constituents into complex-structured supramolecular entities that can serve as building blocks for functional and bio-inspired materials. Moreover, progress in understanding the physics underpinning the evolution of the structure and properties in multiphasic materials has established a foundation for the design of multicomponent materials in which individual constituents autonomously organize into superstructures with tailored properties. To harness the potential of polymeric hybrid materials, an interdisciplinary effort that draws on and reflects the interplay between the synthesis, processing, structure, and performance of these advanced materials is required.

### Biomimetic Design

There is abundant literature describing the increasing number of biomaterials for applications in periodontal regeneration.^48^ Indeed, the development of biomaterials that can be applied for the purpose of periodontal tissue regeneration has been tremendously widened over the last decades. However, there are still some challenges to be addressed, which include the controlled and coordinated release of biological molecules. Along with the advancements made in nanotechnology and associated nanobiomaterials, several other fields of biomaterial engineering are being explored. For example, by utilizing intelligent design strategies, some of these current limitations are expected to be addressed.^47^ Intelligent biomaterials are envisioned to both sense and respond to (external) stimuli. The main aim of intelligent biomaterials is to achieve not only an appropriate timing and quantity, but also continuous administration.

### Bioprinting

One of the most sophisticated applications of additive biomanufacturing involves the fabrication of scaffolds. For the specific requirements of periodontal regeneration, multiphasic scaffolds have significant advantages as they facilitate compartmentalized tissue healing.^24^^,^ 35 While the strategy appears promising, the approach needs to be optimized and tested into large animal studies and eventually in human clinical studies. By taking advantage of the power of additive (bio)manufacturing, exciting developments in the field of regenerative periodontology are envisioned. One such development involves biofabrication technologies, such as bioprinting, which refers to printing of all of the components that form a specific tissue, including living cells embedded in matrix materials, to generate tissue analog structures.^18^ While applications of bioprinting of oral tissues are still in early stages, this strategy has displayed interesting results in various pre-clinical studies and seems encouraging, progressing beyond templates and models.^39^

However, for successful clinical translation it is important to develop a road map, which includes studies to receive the required FDA approval and CE-marking at an early stage in the process. In addition to these general approvals, there is an urgent need for guidelines and protocols for standardization in the field of additive biomanufacturing. Subsequently, these manufacturing and engineering standards need to be merged with biological considerations. Above all, it is important to take into account that bioprinting, seen from a translational research point of view, is primarily focused at patient-specific customization. Therefore, one of the biggest challenges will be establishing standardization, while still allowing for patient-specific adjustments.

## Conclusion

Additive biomanufacturing presents a powerful toolbox to enhance current scaffold-based periodontal tissue regeneration. Currently, additive biomanufacturing facilitates the development of multiphasic scaffolds, which mimic the properties and architectural configurations of periodontal tissues. For optimal exploitation of the potential of additive biomanufacturing, biomaterial characteristics play a pivotal role. By developing advanced biomaterials that influence the biological environment in a controllable manner, patient-specific treatment options with predictable clinical outcomes are envisioned.
